# Longitudinal changes in objectively-measured physical activity and sedentary time among school-age children in Central Texas, US during the COVID-19 pandemic

**DOI:** 10.1186/s12966-022-01299-9

**Published:** 2022-05-19

**Authors:** Leigh Ann Ganzar, Deborah Salvo, Katie Burford, Yuzi Zhang, Harold W. Kohl, Deanna M. Hoelscher

**Affiliations:** 1 Michael & Susan Dell Center for Healthy Living, The University of Texas Health Science Center at Houston (UTHealth) School of Public Health, 1616 Guadalupe, Austin, TX 78701 USA; 2 Prevention Research Center in St. Louis, Brown School, Washington University, 1 Brookings Dr, St. Louis, MO 63130 USA; 3 Department of Kinesiology and Health Education, The University of Texas at Austin, Austin, TX 78712 USA

**Keywords:** Physical activity, Sedentary activity, COVID-19, Social cohesion

## Abstract

**Background:**

Most available evidence on the effects of the COVID-19 pandemic on child movement behaviors is from cross-sectional studies using self-report measures. This study aimed to identify change trajectories and their associated factors for objectively-assessed physical activity and sedentary time among an ethnically and socioeconomically diverse sample of school-age children from Central Texas, U.S.A., during COVID-19.

**Methods:**

Pre- (Sept. 2019 – Feb. 2020) and during- (Oct. 2020 – March 2021) COVID-19 physical activity and sedentary behavior data were collected for school-age children (8–11 years) enrolled in the Safe Travel Environment Evaluation in Texas Schools (STREETS) cohort study. Daily time spent in moderate- to vigorous-intensity physical activity (MVPA) and sedentary time were assessed using GT3X-wBT Actigraph accelerometers. Parent surveys were used to assess socio-ecological factors. Latent class linear mixed models were used to identify change trajectories of MVPA and sedentary time. Logistic regression models were used to assess the association between socio-ecological characteristics with physical activity and sedentary time change trajectory groups.

**Results:**

There was a significant decrease in mean daily MVPA (− 9.4 mins, SD = 18.54) and an increase in sedentary behavior (0.83 hrs, SD = 1.18). Two trajectory groups were identified for MVPA (‘decrease MPVA’ and ‘maintain high MVPA’), with the majority (82.1%) being in the ‘decrease MVPA’ group. Three trajectory groups were identified for sedentary behavior (‘moderate increase sedentary, ‘steep increase sedentary,’ and ‘decrease sedentary’), with most children (78.5%) being in the ‘moderate increase’ group. Girls had significantly lower odds of being in the ‘maintain high MVPA’ group than boys (OR = 0.27, 95% CI = 0.11, 0.61). Children living in neighborhoods with higher perceived social cohesion had significantly higher odds of being in the ‘maintain high MVPA’ group (OR = 1.22, 95% CI = 1.06, 1.41), while those in neighborhoods with higher social cohesion had lower odds of being in the ‘decrease sedentary’ group (OR = 0.86, 95% CI = 0.74, 0.99).

**Conclusions:**

Declines in physical activity and increases in sedentary time among most school-age children during COVID-19 in a socioeconomically and ethnically diverse U.S. sample, were observed in our study, especially among girls. These findings highlight the need to counteract the short-term negative changes in movement behaviors in response to COVID-19 among children.

## Background

Increasing physical activity and reducing sedentary time is a critical public health priority for school-age children globally [1, 2]. The benefits of regular physical activity and low levels of sedentary behavior for children of this age include maintaining healthy weight and the prevention of the onset of obesity later in adolescence and adulthood [3, 4], the prevention of cardiometabolic risk factors [5], and healthy motor and cognitive development [6]. Despite these benefits, in the United States (US), physical inactivity among school age children is alarmingly high, with only 24% of US children ages 6–17 meeting recommended guidelines of 60 minutes of moderate-to-vigorous intensity physical activity (MVPA) daily [7]. Movement behaviors in children also include sedentary behavior. Though current guidelines recommend to limit screen time, an important indicator of sedentary behavior, to less than 2 hours within a 24-hour period, only 5% of US adolescents met all movement behavior guidelines [8]. Within the context of an obesity epidemic in a country in which the leading causes of death are cardiovascular disease and cancer, increasing physical activity and decreasing sedentary time is critical [9].

The SARS-CoV-2 (COVID-19) pandemic and the resulting measures taken by governments disrupted the way in which people across the world live, work, study, travel, and play [10]. The space-use and social interaction restrictions imposed to mitigate the spread of COVID-19, such as social distancing, school closures, and restricted or fully prohibited organized activities, significantly impacted children’s opportunities to meet movement behavior guidelines [11]. Multiple studies, including those from various countries and those focusing on different age groups, have consistently documented declines in physical activity and increases in sedentary behavior during the pandemic [12]. Given the importance of physical activity to children’s health, there remains a need to objectively measure the effect of the pandemic on children’s levels of physical activity and sedentary behavior, and to characterize group-level disparities [13].

Understanding the socio-ecological determinants of changes in movement behaviors during COVID-19 is important to addressing potential disparities. There is some preliminary evidence on the potential determinants of physical activity and sedentary time during the COVID-19 pandemic at multiple-levels of the socio-ecological model [14]. Findings from cross-sectional research, derived from self-report, indicate that at the individual-level, it appears that boys and younger children tended to engage in more physical activity compared to girls and older children, respectively, during COVID-19 [15–17]. Children with high physical activity levels prior to the pandemic also appear to have maintained their physical activity levels during the pandemic [18]. Children with parents who provided greater support for physical activity and who lived in two-parent households have been reported to have higher physical activity levels during COVID-19 than their counterparts [15, 19]. Conversely, lower physical activity levels have been observed during COVID-19 among children whose parents work from home [20]. Lastly, environmental factors are associated with greater physical activity during the pandemic among children, including: living in homes with more outdoor space, living in houses rather than apartments, and living in rural versus urban areas [20–24]. For sedentary behavior, older children and girls have been found to have higher sedentary time during the pandemic across studies, relative to younger children and boys, respectively [19, 25].

Although these cross-sectional, self-report-based studies provide important insights, they have some inherent limitations. Indeed, the lack of studies examining the impacts of COVID-19 on children’s movement behaviors using longitudinal designs, drawing socioeconomically- and racially/ethnically-diverse samples, and using objective physical activity measures, is notable [12, 26]. To date, while there is longitudinal evidence of physical activity changes in young children [27], only one study of school-age children in the US has assessed pandemic-related changes in movement behaviors longitudinally and with objective measures, though this study had a small sample size and did not incorporate subject-to-subject variation [28]. Only one non-US study, with a small sample size, longitudinally measured children’s physical activity and sedentary time changes during the COVID-19 pandemic using accelerometry, and found that among 64 Dutch children (aged 7–12 years), sedentary time was on average 45 min/day higher during May 2020 of the COVID-19 pandemic and MVPA decreased by 17 min/day [29]. As of May 2020, 84.4% of the children in the sample spent less time in MVPA compared to May 2019. However, this study from the Netherlands lacks generalizability to more racially- and ethnically-diverse population groups.

The primary purpose of this paper is to identify pre-COVID-19 to during-COVID-19 change trajectories for objectively-measured physical activity and sedentary time among a racially and socioeconomically diverse sample of school-age children from Central Texas, U.S.A. Additionally, we used a socio-ecological approach to identify individual, familial, social, and built environment factors associated with belonging to different pre-COVID-19 to during COVID-19 physical activity and sedentary change trajectory patterns.

## Methods

### Design and participants

Data for this study came from the Safe Travel Environment Evaluation in Texas Schools (STREETS) study, a five year natural experiment that assesses the health, behavioral, and psychosocial impacts of Safe Routes to School infrastructure changes in Austin, Texas [30]. The STREETS study enrolled elementary school students in a quasi-experimental cohort, with a three-year follow up. The present study uses baseline measures collected during the 2019–2020 school year (pre-COVID-19) and follow-up measures during the 2020–2021 school year (during COVID-19).

The study participants were 3rd and 4th grade students (aged 8–11 years) enrolled in the STREETS cohort at baseline and their parents. Participants were recruited through their schools using flyers, announcements, and electronic communication during the 2018–2019 and 2019–2020 school years. Inclusion criteria for participating in the cohort study were: 1) enrolled in 3rd grade at a participating STREETS cohort school, 2) resided within a 1-mile Euclidean buffer (straight line) of the school, 3) ability to engage in physical activity without significant restrictions, and 4) both parent and child able to complete a written survey in English or Spanish. Parents provided informed consent, and children gave written assent to participate in the study. All study procedures were approved by the UTHealth Committee for the Protection of Human Subjects (HSC-SPH-17-0638), and by the evaluation and research departments at participating school districts.

Baseline measures were conducted during the 2019–2020 school year, prior to the COVID-19 school closures in March 2020. Research staff gave participants study materials during the school hours, and returned eight days later to collect the materials and administer the survey to the children. Parents completed the survey at home and returned the survey to school with the child. At follow up, during the 2021–2022 school year, study materials were mailed or dropped off at participants’ houses, then mailed back to the study team 8 days later. Criteria for inclusion in this study were having valid physical activity data and survey data at both time points.

### Measures

Physical activity and sedentary time were measured using Actigraph GT3X-BT accelerometers (Actigraph Corp. Pensacola, FL). For each study assessment time-point (baseline and follow-up), participants wore waist-worn belts with accelerometers for 7 days during waking hours. Data were recorded at 15-second intervals, and non-wear time was categorized using the Choi (2011) algorithm [31]. Valid wear time for this study was considered to be at least three total days with at least 10 hours of wear-time, and this study did not require a valid weekend day to be included [32]. Evenson population specific cut points for children were used to derive the average minutes of MVPA per day and average hours of sedentary time per day (sedentary: < 100, light physical activity: > 100, moderate physical activity: ≥2296, vigorous physical activity: ≥4012) [33].

Baseline socio-ecological characteristics of participants were measured using parent self-report. Child age and gender were parent reported on the STREETS consent form. Race/ethnicity were reported on the parent survey. Family level characteristics were reported from the parents at baseline, including number of children living in the household (numeric) and parental education attainment (binary: high school equivalent or less vs. above high school equivalent). Parents also answered items related to independent mobility of their children, and were asked whether they would allow their children to walk to recreational or open spaces without an adult and whether they would allow their child to play on streets, playgrounds or parks without an adult [34]. Responses to the independent mobility items were binary (yes/no). School attendance type (binary: in-person/virtual) was assessed by asking parents how their child attended school over the previous seven days. Participants who were home-schooled, attended school virtually by themselves, or attended school virtually in a pod were classified as virtual.

Informal social control and neighborhood social cohesion were measured using two validated scales of five items each [35]. To assess informal social control, parents were asked how likely it was that their neighbors could be counted on to intervene in response to following situations: “Children were skipping school and hanging out on a street corner;” “Children were spray-painting graffiti on a local building,” “Children were showing disrespect to an adult,” “A fight broke out in front of their house, “and “The fire station closest to their home was threatened with budget cuts” (indicating an intention to intervene on behalf of the neighborhood to cuts in public services, like fire response resources). Responses were on a 5-point Likert scale of likelihood (very unlikely to very likely). For social cohesion, parents were asked their level of agreement with the following statements: “People around here are willing to help their neighbors,” “This is a close-knit neighborhood,” “People in this neighborhood can be trusted,” “People in this neighborhood generally don’t get along with each other,” and “People in this neighborhood do not share the same values.” Responses were based off a 5-point Likert scale of agreement (strongly disagree to strongly agree), and the last two items were reverse coded. Scores for each scale were summed to create a continuous variable ranging from 0 to 25, with higher scores indicating higher informal social control (Cronbach’s alpha = 0.92). and social cohesion (Cronbach’s alpha = 0.71).

Perceptions of the neighborhood environment was assessed using four items; parents were asked about the availability (none, some or many) of safe road crossings and sidewalks in their neighborhood (Cronbach’s alpha = 0.81, ICC = 0.79), and responses were dichotomized into low and high categories (low = “none”; high = “some” or “many”) [36]. Perceived safety from crime and safety from traffic were assessed by asking parents how concerned they were about crime and traffic in their neighborhoods, and the response options were on a 4-point Likert scale [37]. Reponses were dichotomized into low and high categories (low = “concerns me little” or “not a concern”; high = “concerns me somewhat” or “concerns me greatly”).

### Statistical analysis

Descriptive statistics, including means and frequencies, were calculated for the participants’ movement behavior and socio-ecological characteristics. This study used a complete case analysis, and a sensitivity analysis was performed to determine differences between the composition of the final analytic sample and to baseline participants lost to follow up or with missing data on key variables. The mean change in daily minutes of MVPA and daily hours of sedentary time were calculated, and paired t-tests were used to assess differences in MVPA and sedentary time between time points. Latent class linear mixed models were used to account for subject-to-subject variation and identify change trajectories of MVPA and sedentary time across time points (pre-COVID-19 to during-COVID-19). Separate latent class models were used for each outcome variable, and models were run for 1–5 groups for each outcome. Model selection was conducted by looking at information criteria (lower AIC and BIC indicated better fit), entropy (higher entropy indicated better fit), and the adjusted Lo-Mendell-Rubin likelihood ratio test [38]. After selecting the best fitting model for each outcome, we combined groupings of MVPA and sedentary behavior to assess combinations of movement behaviors.

The association between socio-ecological characteristics and belonging to different change trajectory patterns was assessed using logistic regression. First, unadjusted models were run for each socio-ecological characteristic and each outcome (separate models for physical activity change trajectories, and for sedentary time change trajectories). Next, partially-adjusted models were run, by including each independent variable of interest plus basic sociodemographic variables (age, sex, race/ethnicity) as covariates. All analyses were run in RStudio (Version 1.4.1717), and the latent class linear mixed models were run using the *“lcmm”* package [39].

## Results

### Sample characteristics

At baseline, 432 children were included in the study. At follow-up, 249 (57.6%) participants agreed to participate, 52 declined (13.1%), and 97 (24.4%) provided no response to contact attempts. The final analytic sample consisted of 168 (38.8%) participants with valid physical activity and survey data for both time points. In sensitivity analyses, participants included in the final analytic sample did not differ significantly to baseline participants who were lost to follow up or had missing data by sex (*p* = 0.18), age (*p* = 0.26), race/ethnicity (*p* = 0.44), or parental education attainment (*p* = 0.14). The average length of time between baseline and follow-up assessment was 11.5 months (SD = 1.9). Descriptive socio-ecological characteristics of the final analytic sample are shown in Table 1.

**Table 1:** Baseline descriptive characteristics of participants in the STREETS cohort in 2019–2020 school year

	*N* = 168
Individual Characteristics	
Sex, n (%)	
Male	74 (44%)
Female	94 (56%)
Age at baseline in years, mean (SD)	8.9 (0.7)
Race/ethnicity, n (%)	
Black or African American	12 (7.0)
Hispanic, Latino, Mexican American, or Spanish Origin	66 (39.5)
White	73 (43.9)
Asian or Other	15 (9.5)
Family Characteristics	
Education level less than high school, n (%)	49 (29%)
Number of children in household, mean (SD)	2.6 (0.9)
Independent mobility rules	
Parents/caregivers allow child to walk to recreational or open spaces without adult, n (%)	103 (61%)
Parents/caregivers allow child to play on streets, playgrounds or parks without adult, n (%)	115 (68%)
Organizational and Social Environment Characteristics	
School attendance during COVID-19, n (%)	
Virtual	90 (54%)
In-Person	78 (46%)
Informal social control, mean (SD)	16.9 (6.0)
Neighborhood social cohesion, mean (SD)	19.2 (3.2)
Neighborhood Characteristics	
Safe road crossings in neighborhood, n (%)	
Low	119 (71%)
High	49 (29%)
Sidewalk availability in neighborhood, n (%)	
Low	83 (49%)
High	87 (51%)
Perceptions of crime safety in neighborhood, n (%)	
Low	78 (47%)
High	87 (53%)
Perceptions of traffic safety in neighborhood, n (%)	
Low	42 (26%)
High	122 (74%)
Movement Behaviors	
Daily minutes of MVPA pre-COVID-19, mean (SD)	48.1 (20.1)
Daily minutes of MVPA during COVID-19, mean (SD)	38.7 (22.7)
Daily hours of sedentary behavior pre-COVID-19, mean (SD)	7.7 (1.1)
Daily hours of sedentary behavior during COVID-19, mean (SD)	8.6 (1.3)
Change in daily minutes of MVPA, mean (SD)	−9.4 (18.5)
Change in daily hours of sedentary time, mean (SD)	0.8 (1.2)
Months between baseline and follow-up measures, mean (SD)	11.5 (1.9)

Notes: *MVPA* moderate-to-vigorous intensity physical activity, Range for informal social control and social cohesion = 0–25

Data on race/ethnicity were missing for 2 participants, data on social control and social cohesion were missing for 1 participant, and data on crime and traffic perceptions were missing for 3 and 4 participants, respectively

### Pre- to during-COVID-19 movement behavior change trajectories

**Fig. 1:**
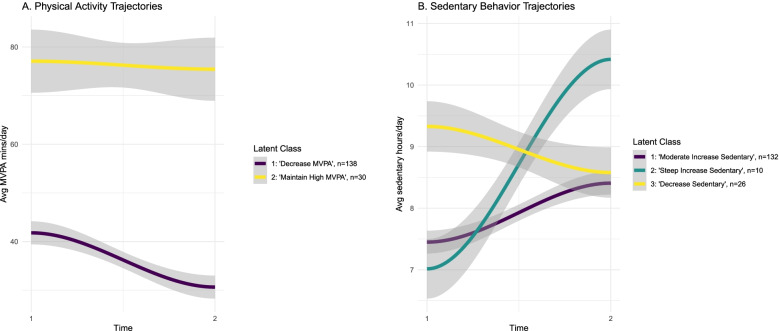
Movement behavior change trajectories in school age children from pre-COVID-19 to during COVID-19

The average change in daily minutes of MVPA prior to the pandemic compared to during the pandemic was − 9.4 minutes (95% CI = 4.81, 14.01, *p* < 0.001), which is a relative change of 17.5%. Based on model fit indices from the latent class linear mixed modeling, a two-group model for MVPA was the best fit (entropy = 0.83, Lo-Mendell-Rubin likelihood ratio (df = 3) = 29.14, *p* < 0.001). Groups were named based on the shape of the trajectories as Class 1: ‘decrease MVPA’ and Class 2: ‘maintain high MVPA’ (Fig. 1). Table 2 shows the sample size and average posterior probabilities for group membership. The majority of participants (82.1%) were in the ‘decrease MVPA’ group, and among this group, the average change in daily minutes of MVPA was − 11.10 minutes (SD = 15.34) from pre-COVID-19 to during COVID-19. Participants in this group had an average of 30.64 minutes (SD = 13.92) of MVPA per day during the COVID-19 time period. In the ‘maintain high MVPA’ group, the average change in minutes of MVPA per day was − 1.66 minutes (SD = 28.06), and during the COVID-19 timepoint, participants in this group had an average of 75.43 minutes of MVPA per day (SD = 18.76).

**Table 2:** Movement Behavior Group Membership From Latent Class Mixed Modeling

	Class Membership ***N*** = 168	Average Posterior Probability
**Physical Activity Group Membership**		
‘Decrease MVPA’ (Class I)	138	0.97
‘Maintain high MVPA’ (Class II)	30	0.88
**Sedentary Time Group Membership**		
‘Moderate increase sedentary’ (Class I)	132	0.93
‘Steep increase sedentary’ (Class II)	10	0.82
‘Decrease sedentary’ (Class III)	26	0.91

For sedentary time, the average change from pre-COVID-19 to during COVID-19 was 0.83 hours per day (95% CI = 0.57, 1.09, *p* < 0.001), and in the latent class linear mixed models, a three group model was the best fitting (entropy = 0.81, Lo-Mendell-Rubin likelihood ratio (df = 3) = 12.49, *p* < 0.001). Group sample sizes and average posterior probabilities of group membership are shown in Table 2. Groups were named based on the shape of the trajectory as Class 1: ‘moderate increase sedentary’ (77.6%), Class 2: ‘steep increase sedentary’ (6.4%), Class 3: ‘decrease sedentary’ (16.0%). The average change in hours of sedentary time per day in the ‘decrease sedentary’ group was − 0.75 hours (SD = 0.71), while for the ‘moderate increase sedentary’ group, the average change was 0.95 hours (SD = 0.80). The average change in daily hours of sedentary time for the ‘steep increase sedentary’ group was 3.40 hours (SD = 0.45).

When a joint group variable was created to assess combinations of changes in MVPA and sedentary time, one participant (0.6%) was in the consistently healthy movement behavior category (‘maintain high MVPA’ and ‘decrease sedentary’). The majority of participants (*n* = 113, 67.3%) were in the consistently unhealthily movement behavior category (‘decrease MVPA’ and ‘moderate increase sedentary’ or ‘steep increase sedentary’). The remaining participants (*n* = 54, 32.1%) fell into the category of a combination of healthy and unhealthy movement behaviors (‘maintain high MVPA’ and ‘increase sedentary’ or ‘decrease MVPA’ and ‘decrease sedentary’).

### Socio-ecological factors associated with movement behavior change trajectories

In logistic regression models to assess the association between socio-ecological characteristics and MVPA group membership, girls had significantly lower odds of being in the ‘maintain high MVPA’ group compared to boys (*p* < 0.001) (Table 3). Additionally, in both unadjusted and adjusted models, participants living in neighborhoods with high perceived social cohesion had significantly higher odds of being in the ‘maintain high MVPA’ group (*p* = 0.005). In the unadjusted model, participants living in neighborhoods with a high sidewalk availability had significantly higher odds of being in the ‘maintain high MVPA’ group compared to participants living in neighborhoods with low sidewalk availability, but this association was no longer significant in the adjusted models.

**Table 3:** Logistic regression of groups of movement behaviors and socio-ecological characteristics

	MVPA Odds of Being in ‘Maintain High MVPA’ Class		Sedentary Time Odds of Being in ‘Decrease Sedentary’ Class	
	Unadjusted OR (95% CI)	Adjusted OR (95% CI)	Unadjusted OR (95% CI)	Adjusted OR (95% CI)
**Individual**				
Age	0.77 (0.43, 1.36)	0.69 (0.36, 1.29)	0.75 (0.40, 1.34)	0.85 (0.36, 1.79)
Sex				
Male	referent	referent	referent	referent
Female	0.27 (0.11, 0.61) **	0.26 (0.10, 0.63)**	1.41 (0.61, 3.40)	1.20 (0.46, 3.29)
Race/Ethnicity				
White, Non-Hispanic	referent	referent	referent	referent
Black/African-American	0.65 (0.10, 2.82)	0.51 (0.07, 2.30)	0.86 (0.04, 5.53)	0.83 (0.04, 5.40)
Hispanic	0.45 (0.17, 1.11)	0.42 (0.15, 1.06)	3.02 (1.19, 8.37)*	2.08 (1.14, 8.14)*
Asian or Other	0.51 (0.07, 2.08)	0.75 (0.10, 3.42)	2.36 (0.46, 9.87)	2.02 (0.38, 8.80)
**Family**				
Parent education level				
High school or less	referent	referent	referent	referent
Above high school	3.15 (1.14, 11.13)*	2.45 (0.63, 10.97)	0.30 (0.13, 0.72)**	0.39 (0.12, 1.17)
Number of children in household	1.32 (0.86, 2.04)	1.48 (0.91, 2.50)	1.15 (0.74, 1.80)	1.20 (0.75, 1.96)
Parents allow child to walk to recreational or open spaces without adult				
No	referent	referent	referent	referent
Yes	0.78 (0.35, 1.78)	0.76 (0.32, 1.85)	1.71 (0.72, 4.41)	1.56 (0.65, 4.22)
Parents allow child to play on streets, playgrounds or parks without adult				
No	referent	referent	referent	referent
Yes	0.88 (0.38, 2.13)	0.89 (0.36, 2.33)	1.23 (0.51, 3.18)	1.28 (0.52, 3.43)
**Organizational and Social Environment**				
School attendance during COVID				
Virtual	referent	referent	referent	referent
In person	1.65 (0.75, 3.72)	1.56 (0.66, 3.74)	1.09 (0.47, 2.49)	0.97 (0.41, 2.27)
Informal social control	1.08 (1.00, 1.17)	1.06 (0.98, 1.17)	0.94 (0.88, 1.01)	0.96 (0.89, 1.03)
Neighborhood social cohesion	1.22 (1.06, 1.41)**	1.21 (1.04, 1.44)**	0.82 (0.72, 0.94)**	0.86 (0.74, 0.99)*
**Neighborhood Environment**				
Safe road crossings				
Low	referent	referent	referent	referent
High	1.05 (0.43, 2.43)	0.87 (0.32, 2.18)	0.83 (0.30, 2.02)	0.90 (0.32, 2.31)
Sidewalks in neighborhood				
Low	referent	referent	referent	referent
High	2.25 (1.00, 5.33)	1.73 (0.70, 4.45)	0.74 (0.32, 1.70)	0.78 (0.32, 1.88)
Concern about crime safety				
Low	referent	referent	referent	referent
High	0.74 (0.33, 1.64)	0.91 (0.39, 2.16)	1.86 (0.79, 4.64)	1.78 (0.74, 4.53)
Concern about traffic safety				
Low	referent	referent	referent	referent
High	2.57 (0.93, 9.15)	2.34 (0.80, 8.56)	1.18 (0.46, 3.42)	1.15 (0.44, 3.41)

Notes: Adjusted odds ratios are adjusted for individual characteristics (age, sex, race/ethnicity)

*p < 0.05, **p < 0.01, ***p < 0.001

For sedentary time, the ‘moderate increase sedentary’ and ‘steep increase sedentary’ groups were collapsed into one group (‘increase sedentary’). Hispanic participants had significantly higher odds of being in the ‘decrease sedentary’ compared to White, non-Hispanic participants (*p* = 0.03). In the logistic regression model, looking at the association between parental education level and sedentary time change trajectory, participants whose parents had above a high school level education had significantly lower odds of being in the ‘decrease sedentary’ group (*p* = 0.002) compared to participants whose parents had a high school level education or less, but this relation was no longer statistically significant in the model adjusted for age, sex, and race/ethnicity. Finally, participants living in neighborhoods with higher social cohesion had significantly lower odds of being in the ‘decrease sedentary’ group (*p* = 0.006).

## Discussion

The purpose of this study was to assess the change trajectories in child movement behaviors from before COVID-19 to during COVID-19 and to examine associations of these longitudinal changes with socio-ecological characteristics of the children. Results showed evidence of significant decreases in physical activity and increases in sedentary time in this sample of school-aged children during the COVID-19 pandemic, consistent with previous longitudinal evidence [28]. This study is the first to describe trajectory groups of movement behaviors using objective measures of MVPA and sedentary time in a diverse sample of elementary school children. The majority of children in this study were categorized in the ‘decrease MVPA’ and ‘moderate increase sedentary’ groups.

Physical activity typically declines with age throughout childhood and adolescence, and the transition from elementary to middle school, which participants in this study were approaching, has been identified as one such critical period in regards to physical activity behavior [40, 41]. Such critical periods of growth and development during childhood are periods during which physical activity and sedentary time behaviors can become habits [42]. There is consistent evidence that physical activity declines over time during childhood and adolescence [43, 44]; a recent systematic review found that in a pooled analysis of yearly relative change in minutes of daily MVPA from age 3 to 18 was − 3.4% [45]. The present study found a mean yearly relative change in minutes of daily MVPA of − 17.0%, indicating that the short-term decline in activity levels in this sample were higher than would be expected without the influence of the COVID-19 pandemic. In addition to maturation, another influence on child physical activity is seasonality, in which typically children are less active during winter months [46]. The average time between baseline and follow-up in this study was 11.5 months, so the two measures were taken at approximately the same time of year, strengthening the evidence that COVID-19 has hindered the development of healthy movement behavior habits among children, as the majority of children in our study decreased physical activity participation and increased sedentary time.

The findings from this study strengthen the existing evidence documenting these same observed patterns of pandemic-related activity behaviors among children globally [12], however, this study also found that children who had high physical activity levels prior to the pandemic also maintained high physical activity levels during the pandemic [18]. Ng et al. (2020) similarly found that Irish adolescents who had prior strong physical activity habits prior to COVID were more likely to report increased physical activity during the COVID-19 lockdown, which may be tied to increased parental support [18]. These data are of additional concern because the ill effects of heart disease and other chronic disease associated with physical activity begin development as early as childhood [47]. Lastly, a large majority of children in our sample did not participate in the recommended 60 minutes of daily MVPA during the pandemic, which may have hindered the cardiometabolic, muscle and bone, and brain health benefits associated with meeting the Physical Activity Guidelines for Americans [48]. In fact, in this study, children in the ‘decrease MVPA’ group had less than half the average daily level of physical activity as the children in the ‘maintain high MVPA’ group during COVID-19 (30.7 vs. 75.4 min/day). The COVID-19 pandemic disrupted the daily routines of children and provided less structured days, and research has shown that children achieve higher levels of physical activity during structured days (like attending school in person) than on weekends or non-structured days [49]. The increases seen in sedentary behavior in this study could be related to the increase in virtual learning and the increase in screen-time [50]. Future research is needed to explore how the short-term negative changes in movement behaviors among children resulted in potentially consequential health effects during the pandemic period, and to determine if children return to pre-pandemic levels of physical activity after the pandemic ends.

This study also identified several socio-ecological characteristics that were associated with healthy movement behavior trajectories. Consistent with previous research, boys had significantly higher odds of maintaining MVPA levels during the COVID-19 pandemic compared to girls [15, 16]. These findings correspond to the consistent and strong evidence that boys engage in more physical activity than girls, regardless of an on-going pandemic [40, 51]. Thus, it may be necessary to implement special interventions that support the physical activity of girls during pandemics or other societal disruptions, including multi-component school-based interventions that target girls only and include components such as dance classes or equipment provision [52, 53].

Additionally, we found that Hispanic children were more likely to decrease sedentary behavior than White, non-Hispanic children. Nationwide, Hispanic parents in the U.S. were less likely to agree that schools should re-open in the fall 2020 semester than White, non-Hispanic parents [54]. Though, in our sample, a majority of Hispanic children attended school in person (53%), compared to 42% of White, non-Hispanic, and this may be contributing the decrease in sedentary behavior due to less screen time.

Our study presented a novel finding that parents with higher perceptions of neighborhood social cohesion had children with a significantly higher odds of maintaining MVPA during COVID-19. The results may be explained by previous literature on social cohesion and physical activity. Specifically, parent perception of neighborhood social cohesion has been shown to be associated with children’s MVPA over time, especially among boys [55, 56]. This may be because social cohesion can influence independent mobility of children and opportunities for physical activity [57]. Parents with higher perceptions of neighborhood social cohesion (i.e., friendliness, trust, shared norms and values, helpfulness) were found to be more likely to permit their child to play outside and travel greater distances independently. Studies have shown that social interactions in green spaces and neighborhood events, such as community art or sports events, can foster social cohesion, highlighting the importance of accessible parks and inclusive programming in neighborhoods for promoting physical activity [58, 59].

We also found that parents with lower perception of neighborhood social cohesion had children that were less likely to decrease sedentary time. Previous studies have shown that neighborhood characteristics, including the social environment, can influence sedentary behavior of children and adolescents [60]. With a lower perception of neighborhood social cohesion, parents may not allow their children to play outside, which results in more time spent inside. Previous research has shown that a higher proportion of light physical activity is done indoors compared to MVPA [61], so children who spent more time indoors during the pandemic may have replaced some of their sedentary time with light physical activity, such as games (board games, made up games with rules), gross motor activity (online videos, dancing, marching, jumping, gymnastics), or chores [62, 63]. Thus, future studies should capture other intensities of physical activity, including light intensity, that may have changed during the pandemic. Overall, our findings suggest that maintaining or increasing neighborhood social cohesion (e.g., community activities, social networks), particularly during a pandemic, may be necessary for encouraging healthy movement behaviors of children.

Although this study provides strong evidence of changes in movement behaviors during the COVID-19 among an ethnically and socioeconomically diverse sample of children, there are several limitations that should be noted. First, the longitudinal nature of this study opens it up to the threat of maturation bias, where study outcomes occur as a result of natural changes over time [64]. Second, although this was the largest objective and longitudinal study among school-age children to date, the sample size was not large enough to run latent class linear mixed models stratified by sex. Additionally, there was a high attrition rate among participants from baseline to follow up, though sensitivity analyses indicated there were no significant demographic differences in our analytic sample compared to baseline. Another limitation to note for this study is the fact that the valid wear time criteria used in this study did not include a requirement for a weekend day to be included, though previous studies have reported that reliability coefficients for accelerometer-based physical activity measures in children are only slightly higher for analyses that include a weekend day compared to analyses for weekdays only [32]. Lastly, there are important determinants of physical activity and sedentary time that were not included in this study which could help to explain the dramatic decline in physical activity and increase in sedentary time. Future research should assess the association of objectively measured built environment variables and physical activity during the COVID-19 pandemic, including land-use mix, residential density, walkability, and access/proximity to recreation facilitate, which have shown consistent direct associations with children’s physical activity [65]. Additionally, this study did not include other potential pandemic-related predictors, such as fewer opportunities to physical activity or the major reported barrier for US adults - concerns about exposure to the virus itself – within analyses [66]. Thus, future research should seek to determine how COVID-19 related barriers and facilitators of health movement behaviors among children. Finally, this study had no control group, but with a societal level exposure like the COVID-19 pandemic, such experimental design was impossible.

## Conclusion

This is the largest existing study to longitudinally measure changes in objective MVPA and sedentary time in school-age children during the COVID-19 pandemic. This study was the first to measure socio-ecological determinants of movement behavior group membership. Results indicated that most children in this sample engaged in significantly less physical activity but more sedentary time. These data support existing research that elementary-school girls were less likely to maintain physical activity levels during compared to boys. Additionally, improving neighborhood social cohesion may be one effective public health strategy for not only maintaining children’s physical activity during the pandemic, but also improving health in general. As there was a marked decline in children’s physical activity and increase in sedentary time, results from this study strongly suggest there is a need to counteract short-term negative changes in the face of another COVID-19 wave, for future pandemic preparation, or for other societal-level disruptors, as children’s current and long-term health is at risk.

## Data Availability

The datasets used and/or analyzed during the current study are available from the corresponding author on reasonable request.

## Abbreviations

MVPA: Moderate-to-vigorous intensity physical activity
SARS-CoV-2: COVID
STREETS: Safe Travel Environment Evaluation in Texas Schools
